# Chronic pulmonary disease is associated with pain spreading and restless legs syndrome in middle-aged women—a population-based study

**DOI:** 10.1007/s11325-018-1673-z

**Published:** 2018-06-04

**Authors:** Zou Ding, Romana Stehlik, Jan Hedner, Jan Ulfberg, Ludger Grote

**Affiliations:** 1 Center for Sleep and Vigilance Disorders, Sahlgrenska Academy, University of Gothenburg, 413 45 Gothenburg, Sweden; 2 Pain Center, Akademiska University Hospital, Uppsala, Sweden; 3 Sleep Disorders Center, Department for Respiratory Disease, Sahlgrenska University Hospital, Gothenburg, Sweden; 4 Circadian Health, Nora, Sweden; 5 Respiratory Medicine, Sahlgrenska University Hospital, 413 45 Gothenburg, Sweden

**Keywords:** Restless legs syndrome, Chronic widespread pain, Hypoxia, Pulmonary disease, Sleep disorder

## Abstract

**Introduction:**

Recent studies suggest an increased prevalence of chronic pain conditions and restless legs syndrome (RLS) in patients with chronic pulmonary disease (CPD). We analyzed the prevalence and risk factors for pain and RLS in a population-based sample of females with comorbid CPD.

**Method:**

Questionnaire-based data from 2745 women aged 18–64 years were analyzed regarding comorbid CPD status (severe bronchitis, emphysema, asthma). Pain status was assessed according to symptoms reflecting severity (Visual Analogue Scale, VAS rating 0–10) and duration and spreading (limited spread or widespread) of pain. A diagnosis of RLS was defined by four validated diagnostic criteria. Anthropometrics and co-morbidities were assessed as covariates in univariate and multivariate analyses.

**Results:**

Widespread pain was overrepresented in women with CPD (44.6 vs. 24.6%, *p* < 0.001). The odds ratio for widespread pain in women with CPD was 1.6 (95% confidence interval (CI) 1.2–2.2, *p* < 0.001) in the fully adjusted model. Severe pain (VAS rating ≥ 7) was more prevalent in females with known CPD (28.8 vs. 15.4%, *p* < 0.001, odd ratio 1.4 (95% CI 1.0–1.9, *p* = 0.029)). The prevalence of RLS was 37.4 and 23.8% in subjects with or without CPD, respectively (*p* < 0.001). In multivariate analysis, CPD was associated with a 30% risk increase for RLS (odds ratio 1.3 (95% CI 1.0–1.7, *p* = 0.04)).

**Conclusion:**

This population-based study identified CPD as an independent risk factor for severe and widespread pain as well as for RLS. Further research addressing pathophysiological mechanisms linking CPD and chronic pain conditions/RLS is warranted.

## Introduction

Pulmonary conditions like Chronic Obstructive Pulmonary Disease (COPD) and bronchial asthma have been associated with chronic pain conditions [1–3]. In a very recent study, up to 77% of patients with COPD suffered from concomitant pain and severity of pain had a significant negative impact on quality of life, mood, and sleep quality [4]. The etiology behind the high prevalence of pain in COPD is incompletely understood but likely to be of multifactorial origin. Potential mechanisms include the modification of sensory input by local hypoxia, fragmentation of sleep, dyspnea, and daytime fatigue [4].

Restless legs syndrome (RLS), also referred to as Willis-Ekbom disease, is characterized by an urge to move often combined with unpleasant sensations in the extremities [5, 6]. Diagnostic criteria have been established in this condition. The clinical management of RLS is well established but the underlying pathophysiological mechanisms of the disorder are still widely unknown. Not only genetic factors but also dopaminergic transmission and dysfunctional central nervous system iron mechanisms have been proposed [6, 7].

Local tissue hypoxia has been shown to alter the pain threshold and may be involved in the development of neuropathic pain [8]. Nocturnal hypoxemia has been linked to pain during sleep [9] and this influence of hypoxia may also include RLS/WED [10]. We have previously identified a strong association between severity of pain, spreading of pain, and RLS suggesting a possible overlap between these chronic pain conditions [11].

Patients with chronic pulmonary disease may express various degrees of systemic and/or local hypoxia. In accordance with the hypothesis stating that pain threshold may be modified by hypoxia, there is data demonstrating a high prevalence of pain and RLS in patients with COPD and sleep-disordered breathing [9, 12–14]. However, the majority of existing evidence is based on data from clinical cohorts. We therefore hypothesized that pulmonary disease may constitute a risk factor for both spreading and intensity of pain and RLS in a female population-based sample.

## Method

### Study aim and previous analyses performed in the study cohort

The initial study hypothesis was based on the clinical observation that RLS prevalence is increased in women suffering from chronic pain, in particular in individuals with chronic multisite pain (Stehlik et al. 2009). The first data analysis in the current questionnaire-based study focused on different chronic pain conditions as a risk factor for RLS prevalence [11]. The main finding was that women with multisite pain had a fivefold increased risk for comorbid RLS compared with women without pain. In order to identify potential underlying mechanisms, a subsequent analysis showed that pain spreading as well as RLS independently compromised sleep quality (amount and continuity of sleep) and contributed to sleep deprivation [15]. The current study focuses on the rather unexplored role of pulmonary disease on pain and RLS prevalence in this cohort.

A brief description of the overall study methodology is given below. The study was approved by the regional ethic review board of Uppsala University (Dnr.2010/124).

### Study population

The survey contained a questionnaire sent out to 10,000 females aged between 18 and 64 years and with a residency in the Swedish county of Dalarna. The study population was stratified for age, but otherwise randomly selected from the population census (approximately 81,000 female inhabitants in the targeted age stratum). The selection was automated and the investigators were not involved in this technical procedure. A study information letter with the heading “Study of the prevalence of chronic widespread pain and restless legs syndrome in females from the Dalarna region” was attached to the mailed questionnaire. A non-responder analysis was performed by telephone interview (*n* = 30) and by analysis of written information provided in returned but otherwise not correctly answered questionnaires (*n* = 70).

### Anthropometric data and comorbidities

Anthropometric data (age (only obtained on a voluntary basis by handwritten comments), height, body weight) and important comorbidities (cardiovascular (CVD), metabolic, and psychiatric disease) were evaluated. Chronic pulmonary disease (CPD) was assessed in the questionnaire and the participant was asked about a physician-diagnosed disorder and/or regular complaints related to the following: a disease of the airway or the lung exemplified by “asthma,” “severe bronchitis,” or “emphysema.” In addition, smoking status (current or no smoking) was determined.

### Classification of RLS/WED symptoms

Standardized and validated criteria were applied [5]. The four characteristic symptoms of RLS, (I) urge to move the limbs and/or dysesthesia, (II) difficulties in resting, (III) worsening of symptoms at rest and improvement by movement, and (IV) worsening at night, were applied in the questionnaire. RLS was assigned if all the four criteria were met (RLS+) and females without any of these RLS symptoms were classified as controls (RLS−) in the subsequent analysis. Subjects with RLS+ were also classified according to RLS symptom frequency (rare, sometimes, often, always).

### Classification of localization, spreading, and intensity of pain

Localization and intensity of pain were assessed by a validated screening questionnaire [16]. In detail, pain spreading was determined for five pain zones (neck, shoulders/arms, upper back region, lower back region, and legs) and three classes of pain spreading were defined: no spreading (0 zones), limited spreading (1–2 zones), and wide spreading (3–5 zones). The intensity of average pain during the last 3 months was assessed using a validated 10-point Visual Analogue Scale (VAS). Severity of pain was rated based on cutoff values on the VAS into no or mild (VAS 0–4), moderate (VAS 5–6), or severe (VAS 7–10) pain [17].

### Classification of subjective sleep quality

All participants were asked to provide information about habitual sleep latency (minutes), sleep duration (hours and minutes), number of nocturnal awakenings, and the desired sleep length. The parameter sleep deficit was calculated from the difference between habitual sleep length and desired sleep duration.

### Statistical analysis

Statistical analysis was performed using IBM-SPSS version 22.0 software (IL, USA). A *p* value of < 0.05 was considered statistically significant. Independent *t*, Mann Whitney *U*, and Kruskal Wallis tests were used to compare normally or non-normally distributed data sets, respectively. Frequency distributions were compared using the chi-square test.

Main statistical analysis constituted generalized linear modeling (GLM) to explore independent predictors of widespread pain (pain reported in three to five body areas), severe pain (VAS scale 7–10), and a diagnosis of RLS (all four criteria fulfilled). The following factors and covariates were included in the analysis: anthropometric data (BMI), smoking status, psychiatric, cardiovascular, and metabolic comorbidity. Known pulmonary disease (defined as above) was used in the model as indicator for impaired respiratory function.

## Results

### Anthropometrics and comorbidities

As previously reported [11], the overall questionnaire response rate was 40.3%. Complete data allowing classification of chronic pulmonary disease was available in 2745 women. A non-responder analysis in 100 women showed that unwillingness to participate, language problems, and non-occurrence of pain and/or RLS were the main reasons for not responding to the questionnaire.

Pulmonary disease was present in 306 participants (11.1%). Women with pulmonary disease had a higher BMI and reported more frequently comorbidities like cardiovascular, metabolic, and psychiatric diseases (Table 1). Current smoking and intake of inhalative respiratory medication (ATC code R03) were more frequent in participants with a diagnosis of pulmonary disease.

**Table 1:** Clinical data of females with and without self-reported pulmonary disease

Parameter	Pulmonary disease (*n* = 306)	No pulmonary disease (*n* = 2439)	Statistics (group difference)
Females (%)	100	100	–
Anthropometric data			
Age* (years)	51.4 ± 10.6	50.3 ± 11.0	n.s.
Height (cm)	164.9 ± 6.4	166.5 ± 6.0	< 0.001
Weight (kg)	76.9 ± 17.4	71.8 ± 14.6	< 0.001
BMI (kg/m^2^)	28.3 ± 6.1	25.9 ± 5.1	< 0.001
Current smoker (%)	20.6	14.6	0.006
Family history of allergic disease (%)	9.8	3.2	0.01
Comorbidities (%)			
Cardiovascular disease	23.4	14.2	< 0.001
Metabolic disease	22.2	11.7	< 0.001
Psychiatric disease	41.2	18.8	< 0.001
Self-reported medication (ATC coded) (%)			
Anti-obstructive inhalative treatment (R03)	36.3	0.2	< 0.001
Analgetic agents (N02)	18.0	12.7	0.011
NSAID (M01)	13.7	9.0	0.008
Dopaminergic treatment (N04)	2.3	0.9	0.025
Pain and restless legs syndrome (%)			
Widespread pain (3–5 areas)^¤^	44.8	25.1	< 0.001
Severe pain**	28.8	15.4	< 0.001
RLS diagnosis fulfilled	37.6	24.1	< 0.001
Frequent RLS symptoms^#^	57.8	40.4	0.001

*n = 1165 subjects with information on age (142 with and 1023 without pulmonary disease)

^¤^Number of reported pain areas in the pain questionnaire (range 0 to 5)

**According to Visual Analogue Scale ≥ 7, mean pain rating during the last 3 months

^#^Defined as symptom frequency “often” and “always” in 710 women with RLS diagnosis

### Pain

**Fig. 1:**
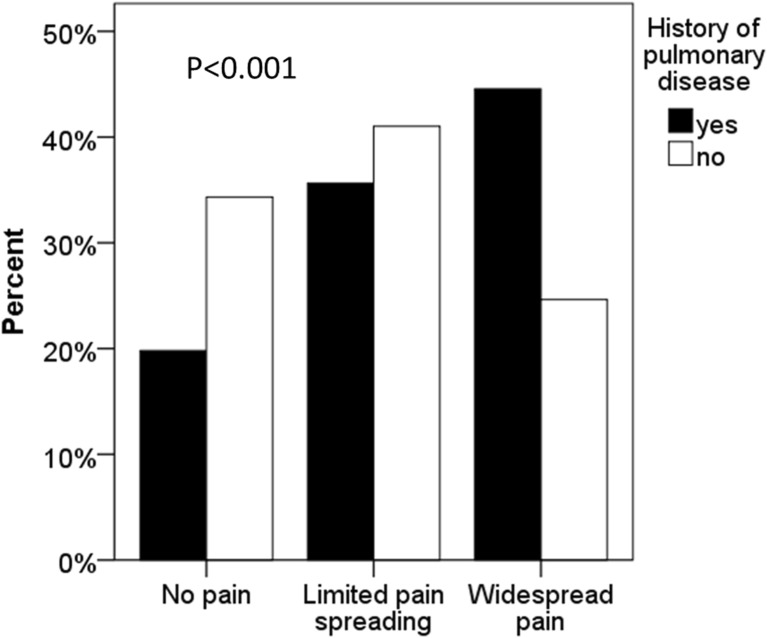
Prevalence of self-reported pulmonary disease in relation to an increasing number of self-reported pain areas (*p* < 0.001). Limited pain spreading, one to two localizations; widespread pain, three to five pain localizations

Females with chronic pulmonary disease had a higher prevalence of widespread pain compared with controls (4.6 vs. 24.6%, *p* < 0.001, Table 1). Conversely, there was a considerably higher prevalence of chronic pulmonary disease in women with widespread pain (Fig. 1). The odds ratio for widespread pain in women with chronic pulmonary disease was 2.4 (95% confidence interval (CI) 1.9–3.1, *p* < 0.001) in the unadjusted model and this increase of risk remained significant also after full adjustment for comorbidities and available anthropometric factors (Table 2).

**Table 2:** Binary logistic regression analysis (*n* = 2745): Odds ratios for pulmonary disease as a risk factor for chronic pain conditions and restless legs syndrome

GLM models	Odds ratio (95% CI)	Significance level for pulmonary disease
Widespread pain		
Model I (unadjusted)	2.4 (1.9–3.1)	*p* < 0.001
Model 2 (adjusted for cardiovascular, metabolic, and psychiatric comorbidities)	1.7 (1.3–2.3)	*p* < 0.001
Model 3 (fully adjusted for comorbidities, smoking, and body mass index)	1.6 (1.2–2.1)	*p* < 0.001
Severe pain		
Model I (unadjusted)	2.4 (1.9–3.0)	*p* < 0.001
Model 2 (adjusted for cardiovascular, metabolic, and psychiatric comorbidities)	1.8 (1.4–2.3)	*p* < 0.001
Model 3 (fully adjusted for comorbidities, smoking, and body mass index)	1.6 (1.2–21)	*p* < 0.001
Restless legs syndrome		
Model I (unadjusted)	1.7 (1.3–2.2)	*p* < 0.001
Model 2 (adjusted for cardiovascular, metabolic, and psychiatric comorbidities)	1.4 (1.1–1.8)	*p* = 0.016
Model 3 (fully adjusted for comorbidities, smoking, and body mass index)	1.3 (1.0–1.7)	*p* = 0.051

*CI* confidence interval

**Fig. 2:**
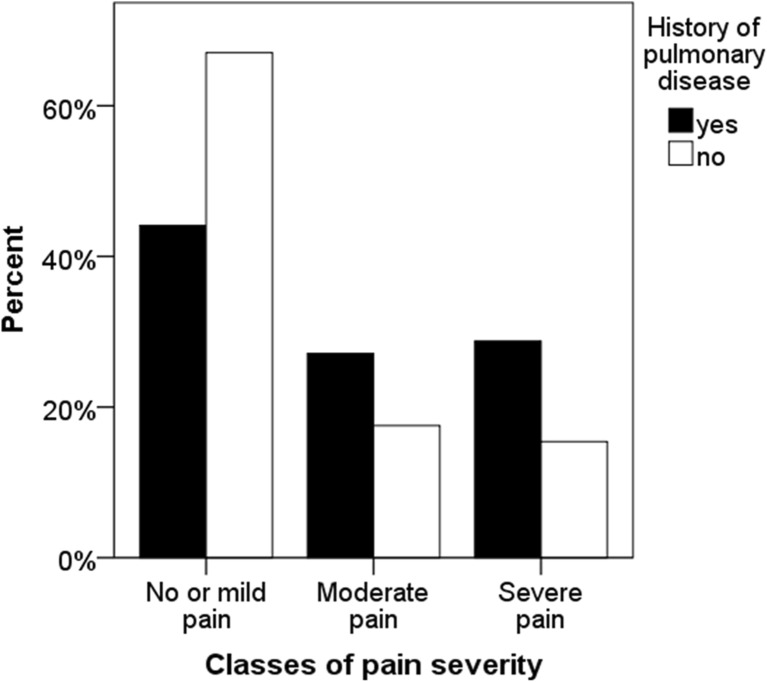
Prevalence of self-reported pulmonary disease in women in relation to increasing self-reported pain intensity (*p* < 0.001). Pain severity was classified according to rating of “average pain sensations during the past 3 months” on the Visual Analogue Scale

**Fig. 3:**
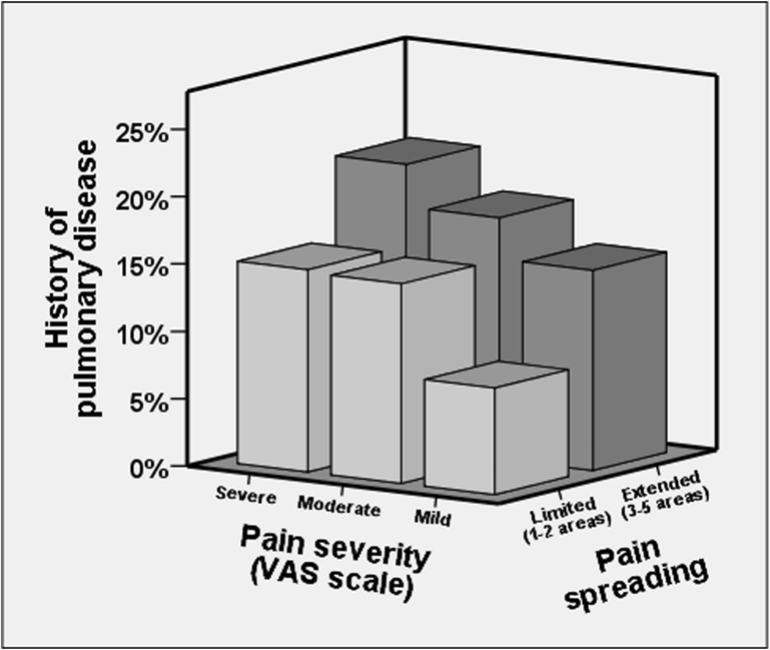
Prevalence of pulmonary disease according to pain spreading and pain intensity (*n* = 1864, *p* < 0.001)

Pain intensity was higher in women with chronic pulmonary disease (Fig. 2) and the frequency of severe pain was almost doubled in women with chronic pulmonary disease compared with those without (Table 1). In fact, chronic pulmonary disease was associated with a 40% higher risk for severe pain independent of comorbidities and BMI in multivariate analysis (Table 2). In an interaction analysis, the prevalence of chronic pulmonary disease increased along with the degree of pain spreading and pain intensity (*p* < 0.001, Fig. 3).

### Restless legs syndrome

**Fig. 4:**
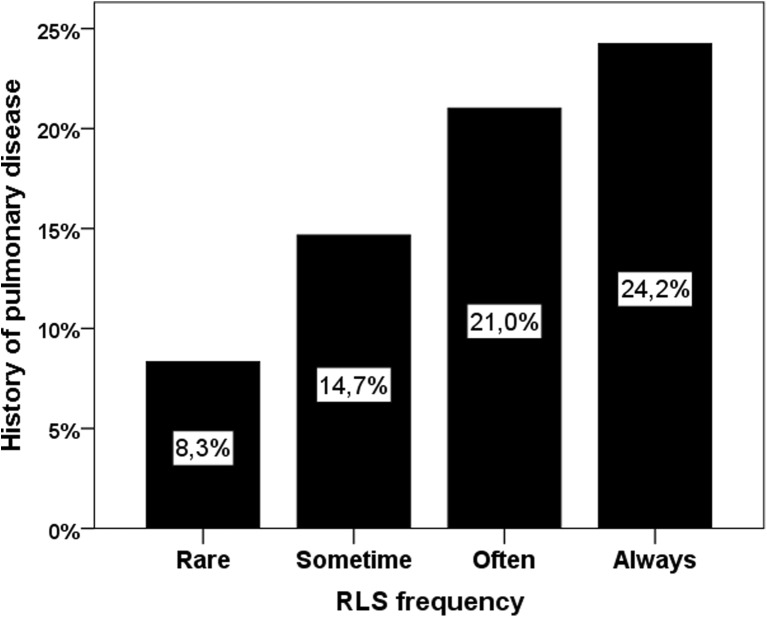
Prevalence of pulmonary disease diagnosis in women with various frequencies of restless legs syndrome (RLS) symptoms (*n* = 710, *p* = 0.001)

The prevalence of RLS was significantly higher in women with chronic pulmonary disease compared with those without a respiratory disorder (37.4 vs. 23.8%, *p* < 0.001, Table 1). Moreover, among women with a RLS diagnosis (*n* = 710), there was a higher prevalence of chronic pulmonary disease in those with a frequent RLS symptomatology (*p* = 0.001, Fig. 4). In GLM analysis, chronic pulmonary disease was associated with a 30% increase of risk for RLS (Table 2). The odds ratio attributable to pulmonary disease was 1.7 (CI 1.3–2.2, *p* < 0.001) in the unadjusted and 1.3 (CI 1.0–1.7, *p* = 0.05, Table 2) in the fully adjusted model. Current smoking, a common risk factor for respiratory disease, was more frequently associated with a RLS diagnosis (32.4 vs. 24.7%, *p* = 0.001) and was identified as an independent risk factor for RLS and pain (*p* < 0.01).

### The influence of comorbid pulmonary disease on sleep quality in pain and RLS

In a subgroup of participants with widespread pain (*n* = 675), severe pain (*n* = 665) and RLS (*n* = 651), we analyzed the influence of comorbid pulmonary disease on subjective sleep quality (Table 3). For all three pain and RLS conditions, sleep latencies were further prolonged in women with pulmonary disease. In addition, sleep deficit was more pronounced in women with comorbid pulmonary disease (Table 3). Women with RLS and severe pain tended to have more frequent nocturnal awakenings.

**Table 3:** Subjective sleep data in females with and without comorbid pulmonary disease in subgroups defined by pain conditions and RLS

Parameter	Pulmonary disease	No pulmonary disease	Statistics (group difference)
Widespread pain^¤^ (3–5 pain areas)			
	*N* = 120	*N* = 555	
Sleep latency (min)	50 ± 49	37 ± 42	0.009*
Sleep duration	381 ± 89	385 ± 77	n.s.
Nocturnal awakenings	3.2 ± 1.9	3.0 ± 1.7	n.s.
Sleep deficit	− 101 ± 80	− 83 ± 72	0.017*
Severe pain (VAS 7–10**)			
	*N* = 115	*N* = 550	
Sleep latency (min)	48 ± 48	36 ± 44	0.006*
Sleep duration	387 ± 92	386 ± 82	n.s.
Nocturnal awakenings	3.2 ± 1.9	2.9 ± 1.7	0.07
Sleep deficit	− 96 ± 87	− 87 ± 76	0.06*
Restless legs syndrome (4 criteria)			
	*N* = 97	*N* = 554	
Sleep latency (min)	49 ± 51	36 ± 47	0.012*
Sleep duration	382 ± 98	394 ± 75	n.s.
Nocturnal awakenings	3.0 ± 1.8	2.8 ± 1.7	0.08
Sleep deficit	− 94 ± 83	− 72 ± 73	0.007*

^¤^Number of reported pain areas in the pain questionnaire (range 0 to 5)

**According to Visual Analogue Scale ≥ 7, mean pain rating during the last 3 months

*Nonparametric test with Mann Whitney *U* test

## Discussion

Our study demonstrates that chronic pain phenotypes characterized by widespread or severe pain and RLS/WED are associated with self-reported chronic pulmonary disease in females. In contrast to most previous studies, our data were obtained in a general population sample of middle-aged females which further supports a link between chronic pain conditions and respiratory disease.

There is only limited data available on the association between pulmonary disease and chronic widespread pain. Hypoxia may modulate pain, and in the Cleveland Family Study, there was an association between minimum nocturnal arterial oxygen saturation and the odds ratio for morning headache and nocturnal chest pain while in bed [9]. Nocturnal hypoxia was also found to be a significant marker of sleep disorders, pain, and fatigue in a community-based cohort of elderly [18]. There is also experimental data supporting a link between hypoxia, pain thresholds, neuropathic pain, and afferent nociceptive nerve traffic in various animal models [8, 19, 20]. Unfortunately, our study does not provide any objective data on oxygenation or pulmonary functional impairment. Another pathomechanisms may relate to sleep quality. In fact, poor sleep is known to reduce the pain threshold and to increase the risk for chronic pain [21]. Pulmonary disease per se may cause nocturnal arousal and prolong nocturnal awakenings caused by dyspnea or periodic airflow limitation. A previous analysis of our data has revealed a strong dose-response relationship between pain spreading and RLS prevalence [11]. The underlying mechanism(s) remain unknown but a possible explanation may involve the augmented sleep fragmentation shown in females with both pain and RLS [15]. In fact, our data points to an impaired sleep function in women with comorbid pulmonary disease including problems to initiate and maintain sleep in a manner that causes extended sleep deficit.

A high prevalence of RLS in clinical cohorts of patients with COPD has previously been described. Spillane reported on the presence of severe RLS in eight COPD patients referred for neurological evaluation [22]. A subsequent study reported an increased prevalence of RLS (29.1%) in 134 patients with COPD [12]. In this study, the RLS diagnosis was associated with the more pronounced hypercapnia and hypoxia occurring in advanced stages of COPD. An even higher prevalence, 36.8%, was reported in a case control study of 87 COPD patients [23]. RLS symptomatology was more severe in those patients with COPD and there was an association with increased daytime sleepiness. Further data demonstrated a RLS prevalence of 54.5% in patients during an acute exacerbation of COPD [24]. In a large population-based sample of males and females from Sweden and Iceland, Benediktsdottir and coworkers found a higher prevalence of RLS in subjects reporting respiratory symptoms, airway obstruction, reduced lung function, or a positive history of current or past smoking [25]. These data are in line with our findings although phenotyping for respiratory function was more elaborated in the Benediktsdottir study. On the other hand, the diagnosis of RLS in our study was based on four validated questions according to contemporary standards and recommendations [5].

The existing epidemiological data is supported by recent experimental studies suggesting that the occurrence of RLS symptoms may be caused by an activation of cellular hypoxic pathways [26]. This study suggested an up-regulation of hypoxic markers including hypoxia-inducing factor 1 alpha, nuclear nitric oxide synthetase, and nitrotyrosine expression in RLS patients. Other subsequent data proposing an hypoxic pathway include a higher iron content in the mitochondria and a reduction in the cytosol of neural cells [7]. The activation of the hypoxic pathway of cellular metabolism has therefore been proposed as a potential mechanism behind dopaminergic dysfunction and RLS symptomatology. Other studies have demonstrated a lower transcutaneous oxygen pressure in the legs of patients with RLS which was reversed by the dopamine agonist pramipexole [10]. These pathophysiological mechanisms in RLS provide a potential link to tissue hypoxia caused by chronic pulmonary disease and may explain the associations demonstrated in the current epidemiological study.

A number of strength and limitations of our study need to be considered. The current study is to our knowledge the first of its kind to investigate a possible association between pulmonary disease and chronic widespread pain conditions in the female general population. The RLS diagnosis and pain characteristics were assessed using well-established and validated questionnaires. More than 650 subjects fulfilled the diagnostic criteria for RLS providing a strong statistical power in our analysis. The response frequency was limited by approximately 40%, a value close to that reported in several recent population-based studies. Uncertainty about the answer from the remaining 60% of women in the target population is a considerable study limitation. In fact, the non-responder analysis suggests a potential oversampling of subjects with pain and RLS symptoms whereas no specific bias was identified for the pulmonary disease classification. However, our study aimed to describe the interaction between chronic pulmonary disease and pain/RLS rather than the prevalence of RLS, pain, or pulmonary disease in this particular population. It is therefore unlikely that study participation bias severely limited our conclusions. Further, language problems were identified as a reason for not participating in the study which comprises a potential risk of underrepresentation of individuals with lower socioeconomic status. As individuals with lower socioeconomic status tend to have higher prevalence of both pain and respiratory morbidities, our results may underestimate the true association of pain conditions and pulmonary disease in the general population. The diagnosis of pulmonary disease was based on self-reported rather than on physiological data from lung function tests or blood gas assessments. However, the 11.1% prevalence of pulmonary disease in these middle-aged women corresponded to what might be expected when comparing with other large-scale cohorts [27]. In addition, the reported frequency of inhaled medications and current smoking history strengthen the classification of pulmonary disease. In fact, the reported frequency of 14.6% of current smoking in our study corresponds closely to data from the female Swedish population published in a report for the years 2006–2013; current smoking prevalence varied between 12.4 and 14.1% [28]. One additional limitation relates to the fact that our findings can only be extended to the female population. Finally, the cross-sectional design of our study does not allow for any conclusions on possible causal relationships between chronic pulmonary disease, RLS, and pain spreading. Further mechanistic and prospective studies are needed to address this issue.

In conclusion, this study is the first to describe a dose-response relationship between pain spreading on one hand and the prevalence of pulmonary disease on the other. In addition, our study supports previous findings of an association between chronic pulmonary disease and RLS. The data calls for future research addressing a potential causal link between impaired oxygenation and chronic pain syndromes including RLS.
